# Myeloid dendritic cells induce HIV-1 latency in non-proliferating CD4+ T cells

**DOI:** 10.1186/1758-2652-13-S3-O7

**Published:** 2010-11-04

**Authors:** VA Evans, S Saleh, EK Haddad, PU Cameron, R-P Sekaly, SR Lewin

**Affiliations:** 1 Monash University, Department of Medicine, Victoria, Australia; 2 CHUM-Research Center, Saint-Luc Hospital, Montreal, Canada; 3 VGTI-Florida, Port St Lucie, USA; 4 Alfred Hospital, Infectious Diseases Unit, Melbourne, Australia; 5 Burnet Institute, Melbourne, Australia

## Background

Resting CD4+ T cells within lymphoid tissues are a reservoir of latent infection; however, in isolated resting CD4+ T cells, several blocks exist that restrict HIV-1 replication. We hypothesize that interactions with dendritic cells (DCs) within lymphoid tissues contribute to the establishment of latency.

## Methods

SNARF-labelled resting CD4+ T cells were cultured alone or with DC for 24 prior to mock infection or infection with a CCR5-using, EGFP-reporter virus. Non-proliferating (SNARF^hi^) CD4+ T cells that were not productively infected (EGFP^-^) were purified five days post infection and: (1) latent infection was reactivated and amplified by co-culturing the sorted cells with mitogen-stimulated PBMC for five days; and (2) gene expression changes were compared in sorted non-proliferating CD4+ T cells cultured in the presence or absence of DCs with or without HIV-1 infection using oligonucleotide microarrays.

## Results

In the presence of DCs, a significant increase in the number of latently infected non-proliferating CD4+ T cells (p=0.01) was observed when compared with resting CD4+ T cells cultured alone. These cells had not entered into the cell cycle as confirmed by the lack of Ki67 expression, although 2% of the DC co-cultured cells did express the early activation marker CD69. Post-integration latency was detected in the non-proliferating CD4+ T cells following co-culture with sorted myeloid (mDC) but not plasmacytoid DC (pDC), which was confirmed using Alu-LTR PCR to detect integrated HIV-1 DNA (11,000 and <300 copies/million cells, respectively). We identified 193 genes that were differentially expressed in the latently infected non-proliferating CD4+ T cells. Observations include the induction of multiple genes associated with cell cycle arrest and the inhibition of HIV-1 transcription.

## Conclusions

Our results suggest a possible pathway for mDC-induced latency in CD4+ T cells in which low levels of cell activation may allow for enhanced HIV-1 integration, but subsequent blocks in transcription and cell proliferation prevent progression to productive infection.

